# Effects of prolonged lock-up time on milk production and health of dairy cattle

**DOI:** 10.1080/01652176.2022.2119622

**Published:** 2022-09-15

**Authors:** L. Papinchak, S. Paudyal, J. Pineiro

**Affiliations:** a Department of Animal Science, Texas A&M University, College Station, TX, USA; b Texas A&M Agrilife Research and Extension Center, Amarillo, TX, USA

**Keywords:** Dairy cattle, cow, bovine, lock-up time, health, cow comfort, milk production

## Abstract

Self-locking feed stanchions provide ease and reduce the amount of time spent handling cattle on free-stall dairy barns. These stanchions assist with routine farm activities such as pregnancy diagnosis, artificial insemination, and various health-related practices. ‘Lock-up time’ refers to the amount of time a cow is restrained in the barn within one day and the producers suggest to keep this duration of time as minimal as possible. This review paper looks at various effects of extended length of lock-up time with regards to milk production, reproductive performance, and dairy cattle health. The objective is to investigate potential effects of extended lock-up time and suggest optimal lock-up time as discussed in the literature. Authors have observed an average lockup time of approximately 1–4 hours per day in the farms in southwest USA. Restraint in self-locking head stanchions for extended period (> 4 h per day) could lead to multiple detrimental effects in dairy cow performance. The focus should be to manage the farm adequately by minimizing the restraint time to less than 4 hours per day, and avoid use of headlocks during late morning and afternoon hours of the summer months. Different studies infer that longer lock-up time presents animals with significant stress situations and represents one of the major issue in dairy industry that needs immediate attention.

## 1. Introduction

Lock-up time refers to the amount of time an animal spends restrained or locked into a head stanchion per day which are located at feed bunks on dairy farms. Dairy cattle are locked up regularly for pregnancy diagnosis, artificial insemination, veterinary-related treatments and examinations, vaccinations, heat detection, and feeding purposes (Arave, Shipka, et al. 1996; Kasimanickam et al. 2018). Headlocks allow a single person to control group of cows which helps in increased labor efficiency during routine herd procedures. The restraint of cows using head locks is a common technique on dairy farms and authors have observed an average lockup time of approximately 1–4 hours per day in the farms in southwest USA. This stretch of time is wide because of variation across dairy farms and between cows within a farm as it depends on the pen size and animal’s position at the feed bunk relative to other pen mates.

Head lock-up as a method of restraint has been found to have varying impacts on an individual animal’s well-being and productive performance within a herd, especially if the system is exploited beyond normal management routine (Arave, Shipka, et al. 1996). Restraint in headlocks for more than 4 h/d is associated with increased aggression in dairy cattle (Kasimanickam et al. 2018). We hypothesize that extended lockup times (>4 hours per day) lead to stress in animals subsequently affecting milk production, reproduction rates, presence of disease, heat stress, lameness, and overall behavior of cattle. This review paper looks at different effects of extended length of lock-up time and tries to identify an optimal lock-up time discussed in the literature.

### 1.1. Management induced stress in dairy cows

Dairy cows often experience stress from management related practices including handling, transportation, social interaction, nutritional deficiencies, heat stress, disease conditions, high stocking density, and lameness. Stressors illicit a response in animals, commonly measured by an increase in cortisol levels, which has the potential to negatively impact endocrine, immune, and neural functions imperative for an animal’s health and productivity (Gwazdauskas 2002).

Repetitive or constant exposure to stressors defined as chronic stress (Trevisi and Bertoni 2009), can lead to significant psychological changes, impacting the severity of the stress response and ultimately leading to altered behavior, decreased immune function, metabolic suppression, and negative effects on growth and production (Kasimanickam et al. 2018). Stress factors initiate multiple pre-pathological and pathological consequences which ultimately reduce animal performance (Trevisi and Bertoni 2009). The variation in stress duration leads to acute and chronic stress situations. Short term stressors, such as hunting and copulating, elicit acute response which are not inherently bad for the animal. These situations serve as triggers for an adrenal response to stress and increase glucocorticoid or catecholamine secretion to help the animal cope with the stressful situation and improve the individual animal’s fitness with energy mobilization (Mostl and Palme 2002). However, chronic stress, such as prolonged lock-up time limits access to water or feed, can exacerbate heat stress, potentially impacts and reduces lying time, as well as alters a cow’s natural time budget. This situation decrease an individual animal’s fitness by causing immunosuppression and atrophy of tissues as a result of prolonged periods of high cortisol concentrations (Mostl and Palme 2002). Additionally, prolonged periods of cortisol secretion have negative impacts on an animal’s reproductive performance and would negatively impact the sustainability of dairy operations.

When cortisol is secreted for a longer duration, the immune system is suppressed, adrenal size increases, spleen size decreases, and epinephrine is elevated, all of which contribute to reduced mammary blood flow and up to a 50% reduction in milk yield (Gwazdauskas 2002). As dairy cows go through repeated exposure to same stressor like head lock-up, they fail to adapt to the stress response which ultimately affects the physiological response to stress (Trevisi and Bertoni 2009). This maladaptation could be responsible for pathologic consequences, which reduce animal welfare and performance (Trevisi and Bertoni 2009). Long-term exposure to stressors leads to chronic stress, which is a risk factor for chronic inflammation. The physiological analyses in a study by Batchelder (2000) demonstrated that stress response and cortisol secretion are related to stressors including longer lock-up times, overcrowding, heat stress, parturition, and lameness. This chronic systemic inflammation affects the performance of the dairy farms among which; decreased milk production and reduced reproductive performance (decreased fertility and lower pregnancy rates) are the most common observations shared by farmers on a dairy that utilize headlocks for prolonged lockup times. Although studies investigating direct relationship of extended lock-up time (> 4 h per day) with markers of chronic stress are scarce, different studies infer that longer lock-up time presents animals with significant stress situations and represents one of the major issue in dairy industry that needs immediate attention.

## 2. Impacts of extended lock-up time

Headlocks are considered necessary evil on the dairy farm. The use of self-locking stanchions is beneficial because of its ease and efficiency in animal handling and worker safety. Lockup stanchions helps reduce competition and aggression at the bunk by ensuring that minimum feed bunk space per cow is available for animals in the pen (Serrenho et al. 2022). However, when this management practice is not executed properly and cows are restrained for extended periods of time (> 4 hours daily), the animals experience varying levels of stress that can be measured using cortisol evaluations (Arave, Shipka, et al. 1996). Animals deprived of lying has been found to take longer to recover from the deviation of overall animal time budget, further exacerbating the situation (Tucker et al. 2021). However, basal blood cortisol is affected by variety of factors including circadian rhythms, sampling, restrain and stage of lactation (Trevisi and Bertoni 2009). Furthermore, during chronic stress situation the basal levels of cortisol is elevated making the measurement of chronic stress very difficult due to the lack of specific tests. Attempts have been made to use indicators like hair and saliva cortisol evaluations, which come with different sets of challenges (Trevisi and Bertoni 2009). Blood fructosamine and hair cortisol are some other indicators of chronic stress currently being studied (Grelet et al. 2022). In addition new tools and technologies like thermography has been explored as non invasive methods to identify chronic stress (Stewart et al. 2007). Most recently, studies have used different acute phase proteins in dairy cows to evaluate wellbeing of the animals (Schmitt et al. 2021). However, research related to the ideal techniques to detect chronic stress associated with head lock-up should be explored.

Prolonged periods in headlocks are associated with restricted forced standing leading to decreased feed intake which lead to altered energy metabolism. Forced standing has been associated with reactivity of HPA axis (Fisher et al. 2002; Tucker et al. 2021). The study by Fisher et al. (2002) identified both ACTH hormone and cortisol levels elevated (11.3 vs 7.6 pmol/L and 106 ± 1.24 vs 101 ± 1.21 nmol/L, respectively) when the cows were deprived of lying for more than 10 hours. Although the study investigated feed restriction as secondary factor, the discussed impact is fully contributed by lockup. Munksgaard et al. (1999) observed similar evidence in their experimental study with young bulls; increased ACTH and cortisol responses due to deprivation of lying for 7 hours. Batchelder (2000) conducted a study looking into cows in headlocks and no headlocks, evaluating them every 15 min for 35 days. Cows in the headlocks for prolonged period demonstrated 3 to 6% reduction in dry matter intake compared to cows without headlocks. Studies have demonstrated that lock-up time for dairy cattle is associated with glucocorticoids secretion ultimately leading to high level of cortisol in blood (Arave, Shipka, et al. 1996, Figure 1). This effect is largely due to limited access of water and feed to the cow when locked up (Relquin and Caudal 1997), reduced lying time, and increased human presence. Although research on commercial dairy farms looking into effects of prolonged lockup is not currently available, there are research indirectly looking into the effects on production and reproduction. The chronically elevated cortisol response have demonstrated adverse effects on milk production, time budget management, and reproduction, which are discussed in detail later in this article. Altered time budget leading to reduced lying time has been associated with reduced sleeping for animals (Tucker et al. 2021), leading to overall disruption of daily rhythm of the cows, who are considered routine animals. Figure 2 illustrates the effects on a multiple areas on a dairy farm due to longer lockup times (>4 h). Physiological response to the stressor as discussed in previous studies are represented in Table 1. In general, extended lock-up time reduces the overall welfare condition of the dairy cows by affecting multiple aspects on the dairy farms.

**Figure 1.**
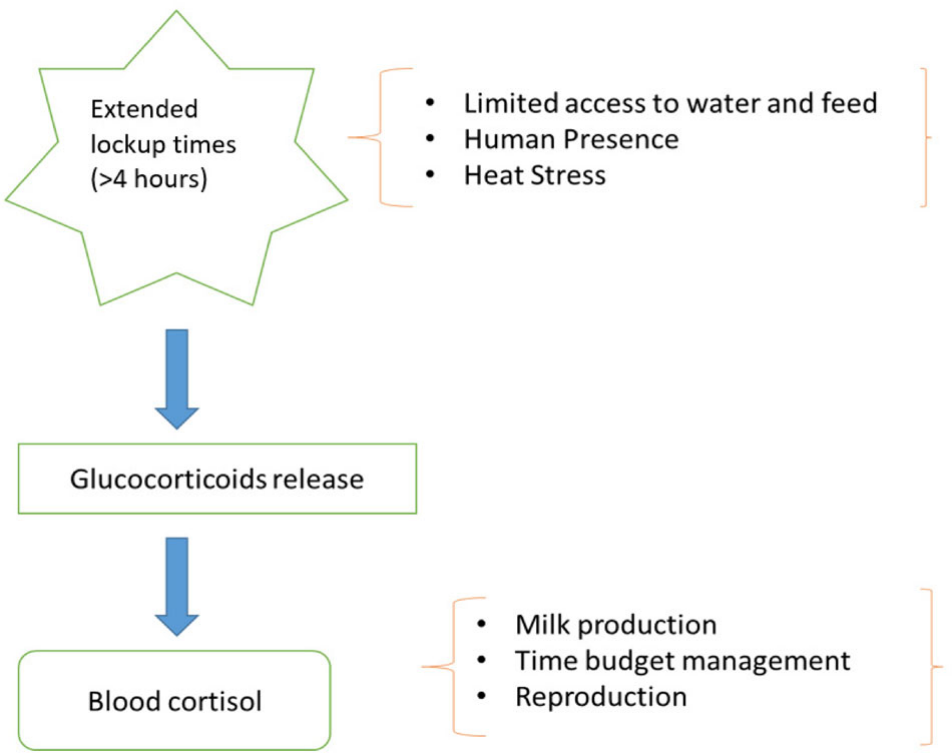
Pathophysiology of stress during extended lock up times (modified after Mostl and Palme 2002; Arave, Shipka, et al. 1996).

**Figure 2.**
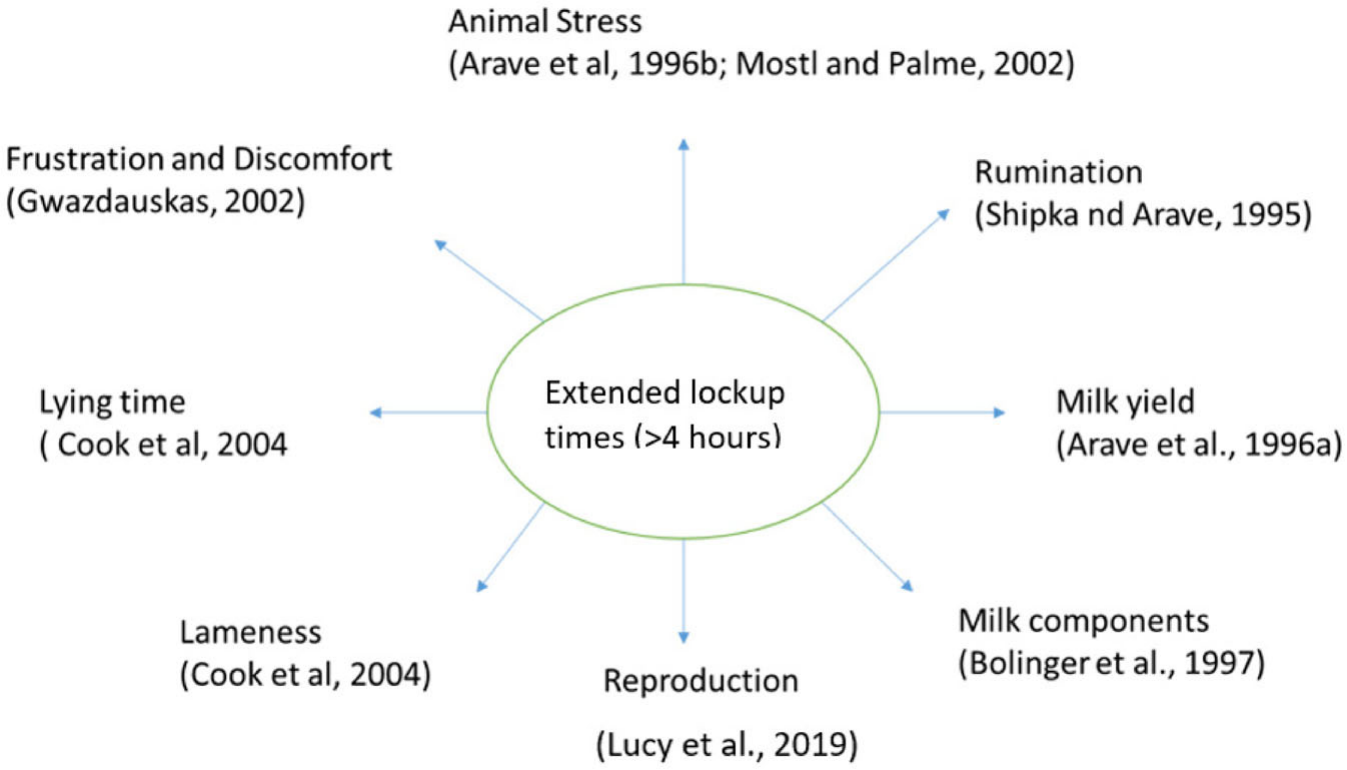
Schematic diagram demonstrating different effects of extended lock up times in dairy cattle.

**Table 1.** Physiological responses to the lock-up times in dairy cattle.

Variable	Lock up time	Response	References
Milk production	2–4 hours	Decreased 2 L/day for 3 days	Cooper et al. 2008
Serum cortisol	4 hours	Increased by 4 ng/mL	Arave, Shipka, et al. 1996
Milk protein	4 hours	Decreased milk protein	Bolinger et al. 1997
Mammary blood flow	–	Decreased blood flow	Rulquin and Caudal 1992
Lying time	2.32 hours	Decreased lying time	Cook et al. 2004
Leg problems	4.31 hours	Increased digital dermatitis, claw horns lesions	Cook et al. 2004
Rumination time	4 hours	Decreased daily rumination minutes	Shipka and Arave 1995
Reproduction	–	Decreased LH due to cortisol	Lucy et al. 2016

### 2.1. Lock-up time and milk production

Reduced milk production is a response observed in cows after lockup time > 4 hours. Mammary homeostasis in the dairy cow is altered because of physiological mechanism involved with the stress response (Giesecke 1985). Stress factors affect the secretory epithelium that promotes altered interstitial equilibrium of the secretory epithelium (Giesecke 1985). We expected similar effects due to stress from a prolonged lock-up time. The altered stress physiology could lead to suboptimal performance of alveoli in mammary gland prompting to a decreased milk yield, higher mastitis incidence and lower milk quality. We also anticipate that these undesirable effects on immune and secretary cells, dairy cows become more prone to mastitis conditions.

Prolonged cortisol secretion has the potential to decrease overall milk yield, but short-term activation of the stress response for up to four-hour period of head-lock restraint was found to have an impact on milk yield, as well as milk fat percentage, somatic cell count, and dry matter intake (Bolinger et al. 1997; Smith et al. 2001; Gwazdauskas 2002; Cooper et al. 2008). Cows deprived of feeding and lying for more than 4 hours reduced milk yield by 2 litres/day for 3 days (Cooper et al. 2008). Milk protein percentage was found to decrease in cows that were restrained from 3.27 to 3.19% (Bolinger et al. 1997). No significant association between mastitis or other health issues were noted in cows restrained for normal duration, except for an increase in viral illness when stress levels were higher due to an increase in blood leukocytes (Bolinger et al. 1997; Gwazdauskas 2002).

A study by Rulquin and Caudal (1992) studied blood flow with relation to lying time and concluded that lying time induced 24% more blood flow to the mammary glands because of cardiovascular homoeostasis due to gravity. Therefore, reduced lying time due to prolonged lock-up time can serve as another explanation for decrease in daily milk yield in dairy cattle.

### 2.2. Lock-up time and time budget management

Dairy cows spend specific amount of time in a day eating, ruminating, and lying down, referred to as time budget, which is altered when they are locked up for extended period (> 4 h). With regards to cow behavior after the lock-up period, various observations have been made over the years. Prolonged lock-up time has the potential to reduce the amount of time allocated to lying per day per cow from the usual time of 12 to 13 h/d (Cook et al. 2004). Lying time is important because it reduces the amount of time a cow spends standing on potentially poor surfaces (Leonard et al. 1996; Cook et al. 2004). However, stall type and cow preference may influence cow behavior and allocation of time within an individual animal’s time budget. Dairy cattle prioritize resting over other behaviors and need to spend 12–14 hours per day lying and 3–5 hours per day feeding (Krawczel and Lee 2019). Overall, we think that alteration of total lying time as a result of prolonged lock-up time could contribute to more time spent standing and helps in development of poor hoof conditions, papillomatous digital dermatitis and claw horn lesions, all conditions commonly associated with lameness (Cook et al. 2004).

The altered time budget due to prolonged lock-up in dairy cows relate to suboptimal performance of these animals. Fifty-three lactating Holstein cows were studied in two studies at Purdue University and Utah State University Dairy for four hours in self-locking stanchions over a four-week period (Shipka and Arave 1995; Arave, Bolinger, et al. 1996). In the first trial, the four hour lock-up period did not impact feed intake, milk production per cow, or SCC per cow, time spent standing, time spent eating TMR, or the blood neutrophil:lymphocyte ratio (Shipka and Arave 1995; Arave, Bolinger, et al. 1996). In the second trial at Utah State University, milk yield was higher during times of no restraint, but other behaviors were observed in the same pattern (Arave, Bolinger, et al. 1996). Additionally, normal herd management lead to an increase in time cows spent lying, self-grooming, ruminating and eating compared to cows in an extended lock-up period (Shipka and Arave 1995).

The altered time budget management due to longer lockup time (> 4 h) affects overall daily cow behavior. More recent behavioral studies determined that cows performed normal oral behaviors with an increase in grooming, eating, and ruminated less frequently, spending more time lying down, and typically exhibited more aggressive behavior after restraint in self-locking head stanchions (Bolinger et al. 1997; Bewley et al. 2001; Gwazdauskas 2002; Kasimanickam et al. 2018). In another study, the authors identified that cows deprived of lying for 2 hours lost their feeding time for next 24 hours whereas cows deprived of lying for 4 hours needed 41 hours to restore the feeding time (Cooper et al. 2008).

Cows with prolonged lockup time also demonstrate a more aggressive behavior. This aggressive behavior was found to be attributed to frustration or discomfort during the restraint period (Gwazdauskas 2002). Aggressive behavior in dairy cows has been associated with lower reproductive performance, including lower conception rates for heifers at first service and in a cumulative comparison where calm heifers were more reproductively successful (Kasimanickam et al. 2018). Additionally, it was found that when cows are deprived of adequate lying time, they increase frequency of some activities such as stomping, repositioning, shifting of weight, becoming restless, and oral stimulation, all of which relate to less desirable animal behavior on dairy farms (Krawczel and Lee 2019).

### 2.3. Lock-up time and transition cow

Extended lockup time is more critical for the cows during the transition period. Because of stress due to calving, cows alter their behavior during the transition period, defined as a period between 3 weeks prepartum and 3 weeks postpartum (Grummer 1995; Huzzey et al. 2005). The transition period is a critical point in the dairy cow’s life, due to the susceptibility to disease, nutritional, physiological, and social changes the animal experiences around the time of calving (Goff and Horst 1997; Huzzey et al. 2005). This period of a dairy cow’s life cycle determines the probability for a successful productive life. However, dairy cows in transition period are more prone to longer headlock time because of the necessity to closely monitor the animal for post calving evaluations and treatment of health disorders. Therefore, during this period, the animal’s state of physiological vulnerability should be considered before implementing a management practice, such as headlock restraint.

Feed intake is very critical in the transition period because of physiological negative energy balance at this stage and this effect could be further exacerbated due to decreased feed intake caused by the prolonged lock-up time. In a study, fifteen transition cows were observed for feeding, drinking, and standing behaviors 10 days before and 10 days after calving to determine how the animal altered its time budget, relative to non-transition cows (Huzzey et al. 2005). The study found that time spent eating declined after calving (87 to 62 min/d), but that animals consumed more meals per day after calving to compensate for this. However, cows spent more time drinking water after calving (6.8 min/d) than before calving (5.5 min/d) (Huzzey et al. 2005). Standing times were observed to be similar pre and post calving (12.3 h and 14.4 h, respectively), with a noticeable increase in standing bouts (21.8) on the day of calving compared to bouts pre and post calving (11.7 and 13.1, respectively; Huzzey et al. 2005). As transition cows are more prone to restraint due to head lockups, these animal behaviors would be further altered. Therefore, stressors placed on the transition cow should be limited and lock-up management routines should be closely monitored for the impacts on altering the transition cow’s time budget and cow comfort.

### 2.4. Lock-up time and lameness

Lameness is a critical issue on dairy farms around the world (Cook et al. 2016) and extended lockup times (> 4 h) have potential to exacerbate the situation. Although studies evaluating direct linkage between lockup times and lameness are not available, there is research suggesting the potential for the effect. Westin et al. (2016) observed cows exposed to narrow feed alley and obstructed lunge space, leading to increased cow standing, were more prone to lameness. In a study with evaluating cows with headlock, cows were observed shifting weight during restraint for more than 4 hrs in the head-lock stanchions, which could be an indication of a threat for lameness in the herd (Bolinger et al. 1997). The increased foot health risks can also be attributed to the abnormal distribution of the cow’s time budget: increased standing time, reduced lying time because of longer lock-up periods, and minimal lying opportunities (Gomez and Cook 2010; Krawczel and Lee 2019). In a study by Cook et al. (2004), the authors found that non lame cows stood 0.73 hours per day, slightly lame cows stood 2.3 hours per day, whereas moderately lame cows stood 4.3 hours per day. Longer lock-up time contributes to deviations from regular daily time budget, indicating variability in lying time and lying bouts that predispose cows to lameness (Ito et al. 2010). Cows that were deprived of lying time, which extended lockup time were found to have increased levels of cortisol in their system, indicating a prolonged activation of the stress response and the potential for negative impacts on the animal’s well-being and physiological stress (Krawczel and Lee 2019). Further exploration of potential direct link between extended lockup time and lameness should be explored in detail.

### 2.5. Lock-up time and heat stress

Dairy cows experience heat stress when the temperature and humidity rise beyond the physiological thermo-neutral zone and we evaluate if the effect is intensified during the head lock up (Cook et al. 2007). The stress due to headlock up can induce manifold negative impacts on the already compromised interrelated biological systems. Heat stress can be detrimental to the dairy cow for various reasons; decrease milk production and milk fat, induce panting as an attempt to perform evaporative cooling that leads to respiratory alkalosis, reduction of dry matter intake, reduced blood flow to the mammary gland, and suppressed reproductive physiological performance and estrus expression (Benjamin 1981; McGuire et al. 1989; Lough et al. 1990; Arave, Shipka, et al. 1996; Ravagnolo et al. 2000; West 2003). Numerous studies have investigated exacerbation of effects of heat stress by the extended lock-up times (> 4 h). Prolonged heat exposure could become problematic when cows are locked up for an extended period of time in extreme climates with high ambient temperatures or high levels of humidity because of the additive effect of the stressors as discussed by Cook et al. (2007). Two consecutive trials were conducted in April and May of 1994 and then in July and August of 1995 to determine how heat factors into extended lock-up time (Arave, Bolinger, et al. 1996; Arave, Shipka, et al. 1996). In each trial, cows were restrained at the feed bunk for 4 hours and serum cortisol was measured (Arave, Bolinger, et al. 1996; Arave, Shipka, et al. 1996). The increase in serum cortisol during lock-up was greater in the summer trial than the spring trial, with means of 24.8 nmol/L and 14.6 nmol/L, respectively (Arave, Bolinger, et al. 1996; Arave, Shipka, et al. 1996). Therefore, extended use of head-lock stanchions on dairy farms in hotter climates is more stressful than in milder climates (Arave, Shipka, et al. 1996). Extended lock-up (> 4 h) has been found to be more detrimental during hotter temperatures than during mild temperatures due to the additive effect of restrain stress and heat stress (Arave, Bolinger, et al. 1996). These evidence suggest that it is more imperative to minimize the lockup time during extreme heat environments in order to reduce the detrimental effects due to the combined effects of these stressors.

### 2.6. Impact on cow social and other factors

Social interactions are an important contributing factor to an animal’s level of stress in the herd and we discuss if they are disrupted significantly due to extended lock up times (Kasimanickam et al. 2018; Lucy et al. 2016). The social interactions include an animal’s behavior at the feed bunk e.g. dominant cows overpower submissive cows. While the study by Endres et al. (2005) indicates that lockup in the feed bunk reduced the aggression at feed bunk and improved access to feed for socially subordinate cows during peak feeding period, the study fails to address the effect of extended head lockup time. Other authors have indicated that restriction at the bunk space can lead to more agonistic behaviors and elicit the undesirable stress response (Gwazdauskas 2002).

Head lock-ups on dairy farms are most often accompanied by human presence in the pen and human interaction with the animals. Human presence and handling is another factor that has been identified to have the potential to induce stress in dairy cattle and negatively impact production. When handled in a brief and gentle manner, milk yield is 20% greater in a cow compared to a more aggressive human interaction (Gwazdauskas 2002). Extended lockups supplemented with aggressive interactions can cause both an acute and chronic or prolonged stress in cattle. Therefore, it is important to reduce the stressor exposure and note a cow’s response to the interaction, as well as make it brief in order to reduce trigger at subsequent behavior (Kasimanickam et al. 2018). Lucy (2019) highlighted that psychological stress have potential for ovarian dysfunction leading to effects in embryonic development and pregnancy. The situation can lead to the activation of the hypothalamic-pituitary-adrenal axis (HPA), causing a responsive release of cortisol that limits luteinizing hormone (LH), released from the anterior pituitary gland, in response to gonadotropin releasing hormone (GnRH) in the hypothalamus (Lucy et al. 2016; Crowe and Williams 2012). Luteinizing hormone is a critical hormone involved in the process of pre-ovulatory follicular development, and ultimately the event of ovulation and formation of the corpus luteum (CL), which is responsible for maintaining pregnancy with the production of progesterone. Although the direct link has not yet been confirmed, extended lock-up times combined with extended human interaction has potential to influence the reproduction performance on dairy farms.

## 3. Appropriate head lockup time

The head lockup time studies are severely lacking in defining the appropriate time without subsequent health and production problems. With the consensus of many dairy producers on stating that less is better, authors have observed dairy farms locking up cows anywhere from 0 to 4 hours. Studies indirectly hint towards indicating that the lock-up time greater than 4 hours per day to be detrimental (Bolinger et al. 1997). Most of the studies seems to take 4 hours as cutoff when evaluating effect of extended locked up (Arave, Bolinger, et al. 1996; Arave, Shipka, et al. 1996; Cook et al. 2008). Further studies to directly evaluate the effect of different time periods on the health and production of dairy cows are warranted in order to be better able suggest the appropriate lockup time.

## 4. Future directions

Self-locking head stanchions continue to be efficient and effective management tools on the farm. These stanchions are intended to be easy to use and comfortable for the cattle, while at the same time improving worker safety and providing determined bunk space per cow (Endres et al. 2005). When used properly, self-locking head stanchions do not significantly affect the overall production on dairy farms (Bewley et al. 2001; Smith et al. 2001). However, research efforts should be directed to identify the threshold lockup duration after which negative impacts would be apparent. Further, research exploring direct impact of lockup time with lameness, health and reproduction is warranted.

## 5. Conclusions

Restraint of cows in self-locking head stanchions for extended period (> 4 h per day) could lead to multiple detrimental effects in dairy cow performance. The focus should be to manage the farm adequately by minimizing the restraint time to less than 4 hrs per day, and avoid use of headlocks during late morning and afternoon hours of the summer months. Research needs to be conducted to quantify the stress response due to prolonged lockup time and provide recommendations for the low impact practices.
